# Will Her Citizens Lead Voluntary Hospital Finance?

**Published:** 1919-11-08

**Authors:** 


### GLASGOW'S UNIQUE OPPORTUNITY.

### Will Her Citizens Lead Voluntary Hospital Finance?

The people of Glasgow are to be envied, for they have a unique opportunity at the moment to make the name of their city famous, and to collect within its borders continuously, at regular intervals, the brightest, ablest, and most famous scientists the world contains, and may contain as the years pass. Glasgow used to have the wish to be recognised throughout the Empire as the second city for its importance, size, wealth, output—everything. Other cities have shown a wish to compete, and if possible supplant, the great city on the Clyde, but to-day the citizens and people of Glasgow and its surrounding districts have the power, if they have the will, enterprise, and public spirit, to lift their city up to a position which will give it the second position in the Empire, providing that between now and December 31, 1919, Lord Lister's work there is worthily perpetuated by the University and people of Glasgow. The citizens, too, have open to them the way through the display of sustained enterprise and united effort to lead in finance and generous wisdom the Voluntary Hospitals throughout the country.

To the writer of this article the charm of the remarkable opportunity now within the grasp of this city on the Clyde is that to accomplish this end and win victory for Glasgow in these enterprises, an opening is given to every citizen to do something individually to raise their city to that of the Second in the Empire. It may then occupy that proud position, possibly, for all time, certainly for so long as Scotland remains a nation of able men and women famous for their enterprise, powers of endurance, industry, application and pride in their city. All of which good things are open to them as citizens to-day.

Lord Lister did more than any other man to save, uplift, and continue life in many types of people, and often to make them strong, or stronger than they were before they came under his treatment. Lord Lister gave them the opportunity—and we may say often, the assured certainty, in God's mercy—of a long and useful life. In a word Lord Lister brought life, and secured life, to men, women, and children to an overwhelmingly larger extent than any other man since the world was made.

In 1860, when Lister was only thirty-three, and had been in Edinburgh only seven years in pursuit of his profession, his great ability and the high reputation that he had acquired there distinguished him as the successor to Lawrie, the Regius Professor of Surgery at the University of Glasgow. The appointment was made by the Home Secretary, approved by Queen Victoria, and Lister removed from the City of the Forth to the City of the Clyde. It was in Glasgow his life's work was done. Here was opened to him a wide field for the exercise of his brilliant abilities; and he received this important appointment while he was still in the full vigour of youth, when he was full of energy and enthusiasm, and when he had sufficient knowledge to fill the position capably, and sufficient experience, both of his profession and of the world at large, to give him confidence and enable him to hold his own. He had studied the art of teaching, had had already much experience in it, and was capable not only of successfully imparting knowledge, but, what is far more important in a teacher, of communicating to his pupils his own enthusiasm and inspiring them with his own ideals. It was an ideal appointment, no less for the University than for the Professor. Here he lived for nine busy, eventful, fruitful, and therefore happy years, and here his great reputation was made.

We recall these facts to the remembrance of the profession and city of Glasgow, especially. There are few now left who remember from personal experience the practice of surgery in the pre-Listerian day, and it is difficult for the surgeons of the present generation to realise what was the state of surgical practice then, and what its results were. Every general practitioner now is a surgeon, and very many general practitioners, probably the great majority of them, are experienced surgeons, who will undertake without hesitation and with success most of the operations of surgery. In pre-Listerian days to open the perinæum was to incur deadly danger. To open the knee-joint was regarded, and justly regarded, as certain death. What operations were performed were left to surgeons, to men who practised surgery and nothing else. The general practitioner rarely used the knife. He was afraid of it. The orthodox operations authorised by tradition, by practice, and by the textbooks were few. It is recorded that it was even possible for a man who had taken honours at a great University, and who had filled all the resident posts at a hospital to which a great medical school was attached, to be afraid to open an abscess, so little was the use of the knife practised. So few operations were performed that one operating theatre supplied the needs of a large hospital of 600 or 700 beds, and even in this theatre operations were performed on only one afternoon of the week, and even on this afternoon sometimes there was but one, sometimes there were none at all. An operation for hernia was a rare event, for it was usually fatal. "Hospital diseases" followed the use of the knife with deadly pertinacity. Again and again the surgical wards of certain hospitals were compulsorily closed, because to enter them as a wounded patient or an operation case was almost certain death. In 1870 Erichsen, whose book on surgery was the standard authority, wrote: "The town free from infection; the hospital saturated by it, to such an extent as to induce surgeons to recommend their patients not to enter it, to compel them to refrain from operating, and, after every attempt

**GLASGOW'S UNIQUE OPPORTUNITY—(*continued*).**

that science and humanity could suggest—every hygienic means employed in vain in the fruitless attempt to eradicate the pestilence from 'the very fabric itself'—to cause the governors, as a last resource, to decide on the demolition of the building, and its complete reconstruction, at a great expense, as the only remedy. But, alas! the new hospitals, built of new materials, were soon as badly infected as the old, or at least the diseases peculiar to hospitals soon appeared in them and clung to them as they had done to the previous building.

It is obvious to us, now that Lister has taught us the lesson, that the infection was not in the fabric of the hospital, but in the patients themselves, and that it was carried, and under the system of practice then in use could not but be carried, from one patient to another. Yet so great was the opposition to the Lister system and his teaching, the truths of which are so obvious to us—now that they have been explained to us—that at the time Lister went to Glasgow each of these truths was received with bitter and determined opposition, almost everywhere throughout the profession. Of what were known as "hospital diseases"—pyæmia, septicæmia, erysipelas, cellulitis, hospital gangrene, and tetanus—the first three were endemic, the last two appeared occasionally only; but, when they did appear, assumed an epidemic spread, and caused a frightful rate of mortality. So the result of an operation was the merest lottery, and could never be predicted. Even an operation which, judging from the size of the incision, appeared to be trivial, might be fatal. Such was the prevailing practice and the prevailing expectation as to results when Lister took up the Professorship of Clinical Surgery at Glasgow. But the work that he did in Glasgow, that has made him and his residence there for all time memorable, demonstrated his to have been an ideal appointment, no less for the University than for the Professor. The nine busy, eventful, and therefore happy years of his Glasgow connection are treasured by Glasgow people of every degree, and here Lister made his great reputation.

**Our subject to-day is Lord Lister and Glasgow, and the opportunity Glasgow has at the moment to perpetuate the memory of Lister's victory and to attach it indelibly to the life, history and glory of the great city on the Clyde.**

### How it Can be Done.

The University of Glasgow has set aside a site and has declared its willingness to re-erect Lister's Ward and Theatre there and to preserve it in perpetual memory of the glorious work done and of the man who did it. Owing to the war Lister's old wards and theatre have been allowed to remain standing, to be used for the accommodation of wounded patients on a portion of the site of the Glasgow Royal Infirmary. There these buildings have proved an obstacle to the perfect hygienic condition and lighting of some of the best wards of this new hospital. They are at present empty, and this site should be cleared without a moment's avoidable delay. It would not take the housebreakers long, nor should the cost be serious, to take down Lister's Ward and Theatre, to clean and repair for building purposes the stone and other material of which the buildings consist, and have them ready for removal, from their present position near the Castle Street entrance, to the site allotted for their reception by, and adjacent to, the University of Glasgow. This work of demolition should be completed by the middle of December, so as to organise and make for ever memorable January 1, 1920, on which day the material of the old Lister workshops should be removed by the citizens of Glasgow themselves to the University site, in proud and loving memory of Lord Lister, the Great Life-saver, his residence in and the great work he accomplished during his nine years as Regius Professor of Surgery at Glasgow. If the whole of the citizens make it their business to do this work themselves the city is sufficiently supplied with the necessary vehicles and means of transit, which should be taken charge of by organised groups of citizens, representing every part of Glasgow and every section of its inhabitants. Thus the world's attention would be attracted and fixed upon the proceedings of this memorable occasion, and scientists and hospital people from all countries, as the years pass, would come in regular sequence to visit and record their names in the Great Book kept in this unique Memorial to the Great Life-saver of the World.

### The Celebration of Victory.

The Lister Memorial might be made the first portion of Glasgow's Celebration of the Nation's Victory in the Great War. To do this worthily, and to make it a feature of surpassing interest attaching to the city of Glasgow for all time, it will surely be in accord with the feeling of the citizens, and with their just pride in their association with the Great Life-Saver, as it is also essential, that the procedure followed shall be worthy of the occasion and be entirely and wholly a Glasgow celebration on the Great Scottish Day of the Year, January 1. Led by the Lord Provost the procession from Castle Street to the University site, if well organised, could be made a most picturesque and memorable sight. The children should have their place, all the trades, professions, establishments, businesses, cathedrals, churches, religious bodies, every form of life and every development in the growth of life-giving features of the city, should each send its quota of representatives and supply banners and other decorative memorial features, so as really to stir the city to its depths, and make it possible for the world to realise, how deep, how all-important, how truly appreciative are the Glasgow citizens of the late Lord Lister, his life teachings and work during his nine years in their midst. It is a great opportunity, and the poetic strain in the Scottish character, as well as its grit, will no doubt carry straight to the

**GLASGOW'S UNIQUE OPPORTUNITY—(*continued*).**

hearts of the citizens how epoch-making this Lister Day can be made, if the leading citizens, with the ablest of the younger members of the community, will join hands under the Lord Provost and bring their brains and knowledge to bear upon the creation of a ceremonial organisation, and the supply of features for the procession, worthy of the great day and of the Great Life-Saver himself.

GLASGOW'S GREAT MODERN HOSPITAL.

The clearance from the site, not only of the Lister buildings, but of the old buildings, including the superintendent's house, the earlier removal of which circumstances have prevented, and of those buildings at present containing cubicles occupied by nurses, will enable the completion of the Great Royal Infirmary to be undertaken. This hospital will then exhibit itself in all the attractive force and beauty of its buildings, and take its place as one of the most interesting, complete, hygienically perfect, and structurally admirable hospital buildings, in the administrative sense, which the country contains. It possesses features which, when the whole of the buildings are completed by the erection at the Castle Street entrance of the remaining portion of the Gatehouse Block, cannot fail to bring interested visitors from all parts of the world to inspect and study it. In 1897 the Lord Provost of Glasgow, Sir David Richmond, promoted a memorial of the Diamond Jubilee of Queen Victoria and formed an executive committee of prominent citizens appointed at a public meeting to raise £100,000. This executive committee ultimately enlarged their scheme by obtaining plans for the reconstruction of the ward blocks, and finally adopted and submitted to the managers the set of plans which had been prepared by Mr. James Miller, F.R.I.B.A., architect, Glasgow. The managers felt, however, that much more was necessary to complete and efficiently equip the great infirmary. They, therefore, elaborated their complete scheme, involving a total expenditure of £500,000, under which the reconstructed Royal Infirmary will accommodate 660 patients. After long and anxious consideration by the managers and staff the plans were subsequently remitted to Sir Henry Burdett and Dr. Donald J. Mackintosh for revision, in so far as the internal arrangements and the hygienic perfecting of the building were concerned, to examine and make suggestions as they thought advisable for their improvement in detail or as a working whole, bearing in mind that the general ground plan of the main ward blocks must not be departed from and that the number of beds provided must not be diminished. In co-operation with the architect, Mr. James Miller, the experts prepared the plans of the new Royal Infirmary, Glasgow, as it is completed, with the exception of that portion of the Admission Block which will provide for the comfort of all the inmates of the existing buildings by the provision of a separate block for noisy and emergency cases. The restriction as to the general ground plan entailed the solving of hygienic problems which are usually absent on account of the adequacy of the site, and its shape, at present provided for a modern hospital. These problems, visitors will find, have been successfully overcome and mastered, so that to-day the Royal Infirmary, Glasgow, is one of the best ventilated and hygienically pleasant buildings devoted to hospital purposes to work in or inspect, which can be found in the world.

The new Royal Infirmary at Glasgow, after ten years' of working experience, when the matters just referred to have been adjusted and the site cleared, will present for the instruction of all expert visitors a great and one of the most interesting of modern hospitals in the world. We have reason to believe that the present managers realise this important fact, and that they are fully desirous to invite the citizens of Glasgow to provide sufficient funds, to do everything that is necessary to complete it to perfection, without a moment's avoidable delay. Having regard to the financial difficulties which beset all voluntary hospitals in this country at the present time, as a temporary but very serious handicap of after-the-war times, we hope that the Citizens of Glasgow may be asked to contribute £150,000, that sufficient money may be in hand to maintain it through the present financial crisis for all the voluntary hospitals in Great Britain, so that the Managers may be enabled to conduct its affairs and defray the cost of bringing it to a state of absolute efficiency, from end to end, without making any further appeal to the citizens for money, for a period of three years from the receipt of the money they now ask for.

Glasgow has solid ground for pride in the Royal Infirmary. Its citizens are men of business whom Providence has blessed for the most part with memorable prosperity throughout the war, and the taking of these matters in hand and the completion of the Infirmary buildings, including a New, Perfected, Up-to-Date Block for the Accommodation of the Nurses (the absence of which constitutes still a grave handicap to the institution, which otherwise would be an excellent example of the best type of modern hospital buildings and administrative thoroughness), with the erection of the Lister Wards on the University site, in commemoration of the great Life Saver of mankind, would, in our judgment, constitute a Grand Celebration of the Peace after the Great War.

Finally, what Glasgow, as a Great Business City undertakes, it can and does quickly and thoroughly carry out. Why should not Glasgow complete everything which is here suggested in Celebration of the Victory and of the Peace its Citizens had so memorable a share in bringing about, by causing the commencement of the building of the Lister Memorial on the University site to be undertaken by January 1, 1920, with the collection and payment to the Treasurers of the Royal Infirmary of the sum of £150,000 by the 31st January, 1920? Such an act would put Glasgow in the position of leadership over all British cities by showing each one of them How to Solve the Financial Problem of their Hospitals in the shortest and completest manner possible to each.

